# Supplementary material for: Quality versus emergency: How good were ventilation fittings produced by additive manufacturing to address shortages during the COVID19 pandemic?
Source: PLoS One. 2022 Apr 21;17(4):e0263808. doi: 10.1371/journal.pone.0263808 (PMC9022824; doi:10.1371/journal.pone.0263808)
Supplement: Attachment — Submitted filename: Reponse to Reviewers.doc [file pone.0263808.s008.doc]

A. **Academic editor**
1. **Please ensure that your manuscript meets PLOS ONE's style requirements, including those for file naming. The PLOS ONE style templates can be found at**
   [**https://journals.plos.org/plosone/s/file?id=wjVg/PLOSOne_formatting_sample_main_body.pdf**](https://journals.plos.org/plosone/s/file?id=wjVg/PLOSOne_formatting_sample_main_body.pdf) [**https://journals.plos.org/plosone/s/file?id=ba62/PLOSOne_formatting_sample_title_authors_affiliations.pdf**](https://journals.plos.org/plosone/s/file?id=ba62/PLOSOne_formatting_sample_title_authors_affiliations.pdf)

*Style requirements have been checked.*

2. **Please include your tables as part of your main manuscript and remove the individual files. Please note that supplementary tables (should remain/ be uploaded) as separate "supporting information" files.**

*Tables have been included into the main manuscript.*

3. **Please ensure that you have an ORCID iD and that it is validated in Editorial Manager.**

*ORCID ID has been provided.*

4. **About suggested references by the reviewer, you could decide either to cite them or not if they are not related to your topic. Moreover, all abbreviations need to be defined at first mentioning. The introduction should be extended, and the novelty should be highlighted compared to the literature review, if there is any. The conclusion part could be rewritten one by one to highlight the obtained results. The number of references should be extended to 35 articles for a better literature review and a better discussion.**

*These modifications have been performed, and the references have been extended to less that 35 articles, but including most of the recent relevant studies on the subject.*

5. **The scale bar should be added on images to know the size of samples. A microscopic image should be added besides Figure 3 to show the quality of samples. In Table 3, what is the dimension of the wall thickness? It is better to add a table for different parameters of 3D-printing for all specimens.**

*Required modifications to figures and tables have been performed. Close views of the devices in Figure 3 have been added.*

B. **Reviewer №1**
1. **Tables 1-3 are missing. So, it is not possible to follow the discussions made by the authors.**

*Tables 1-3 are now inserted into the main text.*

2. **The reasons for using ABS for this application should be described.
   The authors employed five models of industrial leak valves as controls. What are their specifications? What are the essential differences? Summarize this information in a table. Provide a drawing of the ventilation fitting.**

*All these technical data have been added. The drawings of the ventilation fittings are provided in the tables.*

C. **Reviewer №2**
1. **In page 6, starting the line 'we considered (1) in house devices ..... AP-HP 3D- printing platform using FDM and ABS as a raw material,..", please correct to..... ".... using FDM printing technique in which ABS is used as a raw material".**

*This modification has been performed.*

2. **Similarly, please correct.... "(2) in house devices produced........ using polyjet and MED610 as a raw material,............... to... "".... using PolyJet printing technique whereas MED610 is used as a raw material".**

*This modification has been performed.*

3. **PolyJet is a printing technique. Replace polyjet everywhere in the manuscript by "PolyJet'**

*This modification has been performed.*

4. **Please note that many 'articles' are missing in the manuscript, e.g. in Page 6... "......MED610 (Stratasys, Eden Prairie, USA) resin as raw material. " will be."......MED610 (Stratasys, Eden Prairie, USA) resin as a raw material. " (a is missing here**

This modification has been performed.

5. **Finally, I must say that Literature section of the manuscript is very poor. Please note that there are several excellent publications appeared in the last one year in 3D printing and COVID19 crisis. Few more references must be mentioned here, e.g.,
   Tareq et al. https://www.sciencedirect.com/science/article/pii/S0278612520302351
   Andres et al. https://www.sciencedirect.com/science/article/pii/S0278612521000716
   Hannah et al.** [**https://www.sciencedirect.com/science/article/pii/S0278612521001473**](https://www.sciencedirect.com/science/article/pii/S0278612521001473)

*More references (including references provided by Reviewer №2 have been added.*
